# Oxylipins as therapeutic indicators of herbal medicines in cardiovascular diseases: a review

**DOI:** 10.3389/fphar.2024.1454348

**Published:** 2024-12-19

**Authors:** Mengqi Li, Min He, Mengmeng Sun, Yongping Li, Mengyuan Li, Xiaobo Jiang, Yanxin Wang, Hongfeng Wang

**Affiliations:** ^1^ College of Acupuncture and Tuina, Changchun University of Chinese Medicine, Changchun, Jilin, China; ^2^ Northeast Asia Institute of Traditional Chinese Medicine, Changchun University of Chinese Medicine, Changchun, Jilin, China; ^3^ Changchun Sino-Russian Science and Technology Park Co., Ltd., Changchun, Jilin, China; ^4^ Department of Cardiovascular Rehabilitation, The Third Clinical Affiliated Hospital of Changchun University of Chinese Medicine, Changchun, Jilin, China

**Keywords:** herbal medicine, cardiovascular disease, polyunsaturated fatty acids, oxylipins, review

## Abstract

Globally, cardiovascular diseases (CVDs) remain the leading cause of death, and their prevention and treatment continue to face major challenges. Oxylipins, as novel circulating markers of cardiovascular disease, are crucial mediators linking cardiovascular risk factors such as inflammation and platelet activation, and they play an important role in unraveling cardiovascular pathogenesis and therapeutic mechanisms. Chinese herbal medicine plays an important role in the adjuvant treatment of cardiovascular diseases, which has predominantly focused on the key pathways of classic lipids, inflammation, and oxidative stress to elucidate the therapeutic mechanisms of cardiovascular diseases. However,The regulatory effect of traditional Chinese medicine on oxylipins in cardiovascular diseases remains largely unknown. With the increasing number of recent reports on the regulation of oxylipins by Chinese herbal medicine in cardiovascular diseases, it is necessary to comprehensively elucidate the regulatory role of Chinese herbal medicine in cardiovascular diseases from the perspective of oxylipins. This approach not only benefits further research on the therapeutic targets of Chinese herbal medicine, but also brings new perspectives to the treatment of cardiovascular diseases.

## 1 Introduction

The incidence of cardiovascular diseases is increasing worldwide. In 2021, cardiovascular diseases (CVDs) were responsible for the deaths of 20.5 million people globally, accounting for one-third of all global deaths, and standing as the leading cause of human mortality (World Heart Federation, 2023). CVDs remain a major public health issue worldwide, imposing a significant socioeconomic burden. This group of disorders encompasses a variety of heart diseases related to blood flow, circulation, and cardiac function, such as coronary artery disease, heart failure. (Roth et al., 2020). The pathogenesis of CVD involves a complex interplay of genetic, environmental, and lifestyle factors that contribute to the development of key pathological features such as inflammation (Henein et al., 2022), endothelial dysfunction (Xu et al., 2021), and thrombosis (Khodadi, 2020). These pathophysiological processes are mediated by a variety of biochemical and molecular mechanisms, including the dysregulation of lipid mediators (Manke et al., 2022), cytokines (Henein et al., 2022), and cellular adhesion molecules (Troncoso et al., 2021), making them critical targets for therapeutic intervention. As research deepens, researchers have found that cytokines and cellular adhesion molecules are both closely related to lipid mediators, making lipid mediators a key focus in the study of cardiovascular disease mechanisms.

Lipid mediators are important chemical substances in living organisms that can be converted into highly active substances through processes such as fatty acid oxidation. Among them, the oxidation products produced by lipid molecules through endogenous peroxidase and reactive oxygen species pathways are called oxylipins (Nayeem, 2018). These oxylipins play critical roles in modulating various biological processes, including inflammation, vascular endothelial dysfunction, and platelet aggregation (Caligiuri et al., 2017b; Nayeem, 2018). Oxylipins are integral to the regulation of cardiovascular function and pathology, influencing processes such as atherosclerosis (Gleim et al., 2012), hypertension (Gleim et al., 2012), heart failure (Lau et al., 2023) As such, oxylipins represent promising diagnostic tools and target for novel therapeutic approaches aimed at modulating these pathways to improve cardiovascular health (Shearer and Walker, 2018).

Traditional Chinese herbal medicine, which has been utilized for thousands of years in East Asia, is increasingly being integrated into standard biomedical treatments as a complementary or alternative treatment option for CVDs. Interestingly, it has been found that certain herbal medicines, including herbal monomers, extracts, and compound preparations, significantly regulate oxylipin products, their substrates, and the enzymes involved in their pathways (Fu et al., 2018; Zhi et al., 2021). These herbal medicines have also been shown to play an important role in cardioprotection and in slowing the progression of cardiovascular and cerebrovascular diseases in pharmacological studies. This suggests the great potential of Chinese medicines in treating cardiovascular diseases. Therefore, fully elucidating the mechanisms by which Chinese medicines exert cardioprotective effects from the perspective of oxylipins is essential. Such elucidation would facilitate further in-depth studies on the therapeutic mechanisms of Chinese medicines and promote the development of novel therapeutic strategies based on oxylipins, bringing new hope for the treatment of cardiovascular diseases. In this review, we will summarize relevant cellular, animal, and clinical trials, providing a comprehensive overview of the mechanism by which Chinese herbal medicine regulates oxylipins in cardiovascular disease.

## 2 The biosynthesis pathways of oxylipins and their bioactivities relating to vascular and inflammatory regulations

**FIGURE 1:**
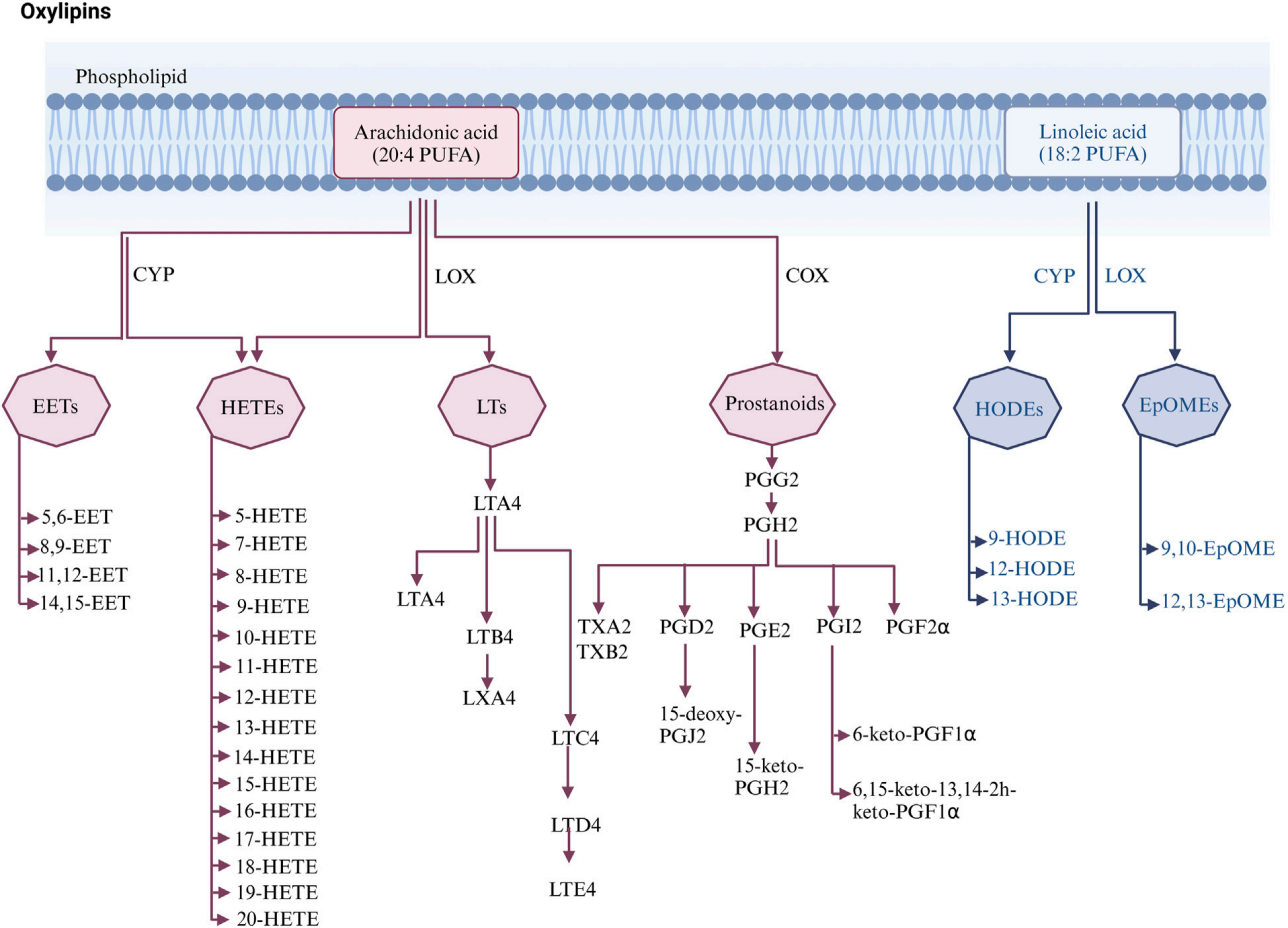
Enzymatic pathways of oxylipins generation.

Oxylipins are bioactive substances formed by the decomposition of polyunsaturated fatty acids (PUFAs) through spontaneous oxidation or enzymatic processes (Nayeem, 2018). Typically, free PUFAs are oxidized into oxylipins either via auto-oxidation, or through three main enzymatic pathways including lipoxygenase (LOX), cyclooxygenase (COX), and cytochrome P450 (CYP450) pathways. Free PUFAs like arachidonic acid (AA) can be converted into epoxyeicosatrienoic acids (EETs) by the CYP pathway, into hydroxyeicosatetraenoic acids (HETEs) by the CYP or LOX pathway, into leukotrienes (LTs) by the LOX pathway, or into prostanoids by the COX pathway; free PUFAs like linoleic acid (LA) can be metabolized into hydroxyoctadecadienoic acids (HODEs) by the LOX pathway, or into epoxymetabolites (EpoMEs) through the CYP epoxygenase pathway (Nayeem, 2018). (Detailed pathways of oxylipins formation are provided in Figure 1). Oxylipin disorder is commonly present in cardiovascular diseases (see Table 1). Oxylipins play diverse regulatory roles (For select examples of oxylipins with cardiovascular bioactive functions, see Table 2), exerting cardioprotective effects and maintaining vascular homeostasis through direct or indirect actions, such as interactions with oxylipin receptors and modulation of inflammation (Hernandez-Saavedra and Stanford, 2022).

**TABLE 1:** Oxylipin changes in cardiovascular diseases.

Diseases	Lipid (↑ Rise; ↓ decline)	Reference
Hyperlipidemia	CYP: ↑ 20-HETE ↓ 14,15-DHET LOX: ↑ LTB4	Caligiuri et al. (2017a)
Hypertension	CYP/LOX: ↑ 5-, 8-, 11-, 12-, 15-, 19-, 20-, HETE LOX: ↑LTD4, TXA2 ↓ PGs Significantly associated with hypertension:11-dehydro-2,3-dinor-TXB2, 12-HHTrE, 295.2279/4.89 (putative eicosanoid), 5,6-EET, Tetranor-12(R)-HETE	Jiang et al. (2015), Xie et al. (2019), Palmu et al. (2020), Feugray et al. (2022)
Thrombosis	COX: ↑ TXA2, TXB2, 6-keto-PGF1α Ratio: ↑ TXB2/6-keto-PGF1α	Dang et al. (2015), Zhang et al. (2020)
Atherosclerosis	COX: ↑ TXB2 ↓ 6-keto-PGF1α Ratio: ↑ TXB2/6-keto-PGF1α	Zhang et al. (2013), Tong et al. (2022)
Coronary artery disease	CYP: ↑ EETs, 9,10-EpOME, 8-HETE, 12,13-EpOME, 8,9-DiHETrE LOX/CYP: ↑ (±)5-HETE, PGE2 ↓ LXA4 Correlation with CAD: PGD2, PGE2, 15d-PGJ2 and 5-HETE negatively correlated with CAD; 13-oxo-ODE positively correlated	Li et al. (2016)
Acute coronary syndrome	COX: ↑ TXB2, 6-keto-PGF1α, TXB2/6-keto-PGF1α, PGE2 CYP/LOX: ↑ 8,9-DiHETrE, 11,12-DiHETrE, 8-HETE, 9-HETE, 11-HETE, 20-HETE, 20-COOH-AA LOX/CYP: ↑ LTB4, 5-HETE, 12-HETE, 15-HETE, 12-HEPE ↓ 9-HODE Associated with ACS: Higher levels of 19-HETE are associated with a better prognosis for ACS Plasma concentrations of 18-HEPE were positively correlated with ACS	Solati and Ravandi (2019)
Myocardial infarction	CYP: ↑ 20-COOH- AA,19,20-EpDPE, 8-HETE, 9-HETE, 11-HETE ↓ 5,6-EET, 8,9-EET, 14,15-EET, 5,6-DHET, 11,12-EET, 14,15-DHET, 16-HETE, 17-HETE, 12,13-EpODE, 12,13-EpOME, 9,10-EpOME COX: ↑ TXB2, TXA2, PGE2, PGF2, 15-Deoxy-PGJ2 ↓ 6-keto-PGF1α, PGI2 ↑↓ PGD2 LOX: ↑ 7-HDoHE, 8-HDoHE, 17-Keto DHA, 5-HEPE, LTB4, 6-trans LTB4, 20-COOH-LTB4, 15-HETE, 12-OxoETE, 15-HPETE ↓tetranor 12-oxoETE, 9-oxoOTrE, 11-HDoHE, 9-HODE, 13-HODE, 9-OxoODE, 13-OxoODE, LXA4 ↑↓ 5-HETE, 12-HETE, 5-oxoETE, 15-oxoETE, 20-HETE Ratio: ↑ TXB2/6-keto-PGF1α	Freeman et al. (2015), Cao et al. (2016), Li et al. (2016), Rocic and Schwartzman (2018), Roman and Fan (2018), Huang et al. (2020), Jiang et al. (2021), Zhi et al. (2021), 2021; Solati et al. (2023)
Myocardial infarction reperfusion injury	CYP: ↑ 20-COOH-AA LOX: ↓ 9-HODE, 13-HODE	Solati et al. (2023)
Heart failure	CYP: ↑ 9,10-EpOME, 12,13-EpOME, 9,10-DiHOME,9,10,13-TriHOME, PGE2, 19,20-DiHDPA, 5,6-DiHETrE,14,15-EpETE, 14,15-DiHETE, 17,18-DiHETE LOX: ↑ 9-oxoODE, 13-oxoODE, 9,10,13-DiHODE, 12,13-DiHODE, 16,17-EpDPE, 15HETE, 15-hPETE, 20-carboxy-LTB4, 9-HOTrE ↓ 8-HOTE, 8-HETrE COX: ↑ 13,14-dihydro-PGF2α, TXB2, PGE2 ↓6-keto-PGF1α Ratio: ↑ TXB2/6-keto-PGF1α RNS: ↑ NO2-LA Automatic oxidation: ↓ 20-HDoHE	Wang et al. (2015b), 2015a; Lau et al. (2023)

**TABLE 2:** Cardiovascular bioactive functions of oxylipins.

Substrate	Lipid	Bioactive functions	Reference
LA	9-HODE	Harms: regulation of stress and inflammation	Kwon et al. (2020)
	13-HODE	Benefits: inhibits platelet adhesion and aggregation	Buchanan et al. (1991), Marmol et al. (1999), Belvisi and Mitchell (2009)
	9, 10-EpOME	Harms: cardiac inhibition, cardiotoxicity, cytotoxicity	Sugiyama et al. (1987), Hildreth et al. (2020)
	12, 13-EpOME	Dual action: Harms: cardiotoxicity, cytotoxicity, induction of heart failure Benefits: improvement of cardiac structure and function	Bannehr et al. (2019), Hildreth et al. (2020), Pinckard et al. (2021), Lau et al. (2023)
AA	5, 6-EET	Benefits: anti-inflammatory; relaxes vascular smooth muscle; affects migration and proliferation of endothelial and vascular smooth muscle cells	Spector (2009), Sudhahar et al. (2010), Tacconelli and Patrignani (2014), Zuo et al. (2021)
	8, 9-EET	Benefits: anti-inflammatory, improves fibrosis and apoptosis, vasodilator	Sudhahar et al. (2010)
	11, 12-EET	Benefits: anti-inflammatory; vasodilator, prevents IR-induced mitochondrial dysfunction, reduces ROS levels	Sudhahar et al. (2010)
	14, 15-EET	Benefits: restoration of IR, cardioprotective effect, vasodilator	Sudhahar et al. (2010)
	5-HETE	Harms: induces inflammation and cardiomyocyte hypertrophy	Nayeem (2018)
	11-HETE	Benefits: inhibits proliferation of human vascular smooth muscle cells	Brinkman et al. (1990)
	12-HETE	Harms: alter vascular tone, induce vascular endothelial growth factor growth, Induction of myocardial fibrosis and hypertension	Buchanan et al. (1998), Stanke-Labesque et al. (2002)
	15-HETE	Dual action: Benefits: inhibits atherosclerosis, inhibits leukocyte adhesion and aggregation, and vasodilates blood vessels Harms: induces myocardial fibrosis, heart failure, and inflammation	Wittwer and Hersberger (2007), Buckley et al. (2014), Hernandez-Saavedra and Stanford (2022)
	16-HETE	Benefits: vasodilatation, inhibition of adhesion and inflammatory response	Reddy et al. (2003), Shoieb et al. (2019)
	17-(R/S) -HETE	Harms: Induced cardiac hypertrophy	Isse et al. (2023)
	19-HETE	Benefits: vasodilator, anti-inflammatory, regulates blood pressure, prevents cardiac hypertrophy	Alonso-Galicia et al. (1999), Zhang et al. (2005), El-Sherbeni and El-Kadi (2014), Shoieb et al. (2019)
	20-HETE	Dual action: Harms: vasoconstriction, pro-inflammatory Benefits: promotes endothelial cell proliferation	Chen et al. (2012), Hoopes et al. (2015)
	5-oxoETE	Harms: trigger myocardial injury	Lai et al. (2021)
	15-oxoETE	Benefits: antihypertensive effect, antioxidant responses, and inhibits pro-inflammatory responses	Snyder et al. (2015), Yu et al. (2023)
	5, 6-DiHETrE	Related to heart failure	Zhang et al. (2016)
	PGD2	Benefits: anti-inflammatory, inhibits platelet aggregation, vasodilator	Cipollone (2008), Yousefnia et al. (2018)
	15-deoxy-PGJ2	15-Deoxy-PGJ2 is a PGD2 metabolite that activates plasminogen activator inhibitor type-1 via PPAR-activation in endothelial cells	Caligiuri et al. (2017b)
	PGE2	Dual action: Harms: increased vascular permeability, immunosuppression, pro-inflammatory, reduction of infarct size, alleviation of neutrophil accumulation in reperfused myocardium Benefits: anti-inflammatory	Nayeem (2018), Hernandez-Saavedra and Stanford (2022)
	PGF2α	Harms: constricts coronary vessels, promotes cardiac dysfunction and hypertrophy, pro-inflammatory, associated with tachycardia, cardiac dysfunction, and cardiac hypertrophy	Hernandez-Saavedra and Stanford (2022)
	PGI2	Benefits: reduces atherosclerosis, prevents thrombosis and atherosclerosis, lowers hypertension, prevents cardiac hypertrophy, vascular remodeling	Yuhki et al. (2011), Hernandez-Saavedra and Stanford (2022)
	TXA2	Harms: promote platelet aggregation, adhesion, vasoconstriction, pro-inflammatory	Hernandez-Saavedra and Stanford (2022)
	TXB2	Harms: hypertension and vascular dysfunction	Hernandez-Saavedra and Stanford (2022)
	LTB4	Harms: pro-inflammatory, promotes vascular endothelial cell adhesion, related to unstable atherosclerotic plaque	Qiu et al. (2006), Das (2021)
	LTC4	Harms: pro-inflammatory, promotes plaque formation and myocardial ischemia	Nobili et al. (2012)
Ratio	TXB2/PGF1α	Harms: promoting platelet activation and inducing cardiovascular events	Caligiuri et al. (2017a)

### 2.1 Epoxyeicosatrienoic acids (EETs) in cardiovascular systems

EETs are synthesized in cardiomyocytes and endothelial cells (Campbell et al., 2017) through the metabolism of AA. Cytochrome P450 enzymes, such as CYP2J2, convert AA into four regioisomeric EETs: 5, 6-, 8, 9-, 11, 12-, and 14,15-EET, which are then rapidly hydrolyzed to the less active dihydroxyeicosatrienoic acids (DHETs) in the presence of soluble epoxide hydrolase (sEH) (Lai and Chen, 2021). EETs have a wide range of cardioprotective effects, including inhibiting cardiomyocyte hypertrophy, ameliorating apoptosis, inhibiting platelet adhesion to endothelial cells, and possessing anti-inflammatory properties (Krötz et al., 2004; Spector, 2009; Tacconelli and Patrignani, 2014). DHETs, as stable metabolites of EETs, reflect the concentration of EETs in circulation. These metabolites contribute to improved vascular endothelial function by increasing the expression of nitric oxide, which has been identified as the main chemical compound of endothelial-derived relaxation factors (Zuo et al., 2021).

In CYP2J knockout (KO) rats, plasma EET levels were significantly reduced, exacerbating myocardial inflammation, hypertrophy, and fibrosis. Additionally, myocardial injury became more severe with age in KO rats, corresponding with significant reductions in 11,12-EET and 14,15-EET levels in the plasma (Zhang Y. et al., 2023). Changes in EET or DHET were also observed in animal models of hyperlipidemia and acute myocardial infarction (AMI). Specifically, levels of 14, 15-DHET decreased in hyperlipidemic mice (Chen et al., 2022); and 5, 6-EET, 8, 9-EET, 14, 15-EET, 5, 6-DHET, 11, 12-EET, 14, 15-DHET showed a clear downward trend in post-myocardial infarction rats (Zhi et al., 2021). In large-scale clinical studies, coronary artery disease (CAD) patients exhibit elevated levels of plasma EETs (Theken et al., 2012) and 5, 6-EET is closely associated with blood pressure status (Palmu et al., 2020).

### 2.2 Hydroxyeicosatetraenoic acids (HETEs) in cardiovascular systems

AA produces medium-chain HETEs, including 5-, 8-, 9-, 11-, 12-, and 15-HETEs, through allylic oxidation in the presence of LOX (Nayeem, 2018). In addition, AA is also metabolized by CYP450 enzymes, such as CYP4A and CYP4F, through allylic oxidation to produce terminal HETEs, including 16-, 17-, 18-, 20-HETE, etc. (Shoieb et al., 2019). Multiple cell types in the heart, including cardiomyocytes and fibroblasts, synthesize and respond to HETEs (Escoubet et al., 1986).

Unlike the effects of EETs, most medium-chain HETEs have pro-inflammatory and vasoconstrictive properties, and their formation can increase cardiovascular dysfunction (Nayeem, 2018; ElKhatib et al., 2023). HETEs also block the synthesis of EETs by RL-14 cells and increase the conversion of EETs to DHETs, affecting EET levels (Maayah et al., 2015). Among the medium-chain HETEs, 12- and 15-HETE are closely associated with the cardiovascular system (Breitbart et al., 1996; Huang et al., 2020). Both are produced through CYP in addition to the LOX pathway. 12-HETE has chemotactic properties, can alter vascular tone, and induce VEGF growth (Buchanan et al., 1998). Levels of 12-HETE were also significantly elevated in patients 24–40 h after acute myocardial infarction (Freeman et al., 2015). 15-HETE has been reported to have anti-inflammatory effects and can be converted to LXs, which play a role in reducing inflammation (Buckley et al., 2014). In large-scale clinical studies, patients with coronary artery disease who experienced an acute myocardial ischemic event within 2 years had elevated levels of 8-, 9-, 11-, 12-, and 15-HETE compared to those without a cardiovascular event (Huang et al., 2020). In hypertensive patients, elevated levels of 5-, 8-, 11-, 12-, and 15-HETE have also been observed (Xie et al., 2019; Feugray et al., 2022).

Among the terminal HETEs, 16-HETE, 18-HETE, and 19-HETE and metabolites of 20-HETE can promote vasodilation (Carroll et al., 1996; Caligiuri et al., 2017b). The main functions of 16-HETE include promoting vasodilation and inhibiting inflammation mediated by neutrophil adhesion (Reddy et al., 2003; Shoieb et al., 2019). The precise role of 20-HETE in the cardiovascular system is complex. Studies have shown that 20-HETE produced by CYP4A is an effective vasoconstrictor in mouse aortas and coronary arteries (Hoopes et al., 2015), but there is also research evidence suggesting that 20-HETE can promote the proliferation of endothelial cells through activation of the vascular endothelial growth factor (VEGF) pathway. In clinical trials, among acute coronary syndrome (ACS) patients, the main abnormalities in oxylipins concentrate in terminal HETEs, with higher levels of 19-HETE associated with better ACS prognosis (Solati and Ravandi, 2019). In AMI rats, levels of oxylipins such as 20-HETE increased, while 16-HETE and 18-HETE showed a decreased tendency post-myocardial infarction (Zhi et al., 2021).

However, it is worth noting that HETEs have a relatively short existence time and can be converted into oxoETE under the action of dehydrogenase. Among various oxoETEs, 5-oxoETE has been shown to have the effect of inducing myocardial injury (Lai et al., 2021); And 15-oxoETE has a hypotensive effect (Yu et al., 2023). In cell cultures, 15-oxoETE activates NRF2-regulated antioxidant responses and inhibits NF-κB-mediated pro-inflammatory responses (Snyder et al., 2015).

### 2.3 Leukotrienes (LTs) in cardiovascular systems

Leukotrienes (LTs) are formed from AA through the 5-LOX pathway, producing unstable epoxide metabolites LTA4, which are then metabolized by leukotriene A4 hydrolase (LTA4H) and leukotriene C4 synthase (LTC4S) into various metabolites like LTB4, LTC4, LTD4 (Hammarström, 1983). LTs play roles in various acute and chronic inflammations. Especially LTB4, which can induce the release of pro-inflammatory cytokines, recruit and infiltrate leukocytes, and promote inflammation (Das, 2021). LTC4 has been reported to be associated with pro-inflammatory activities, plaque formation, and myocardial ischemia (Nobili et al., 2012). In some animal experiments, researchers also observed changes in LTs. For example, serum LTB4 levels were significantly increased in hyperlipidemic rats (Wang Y. Q. et al., 2020). LTD4 showed high levels in spontaneously hypertensive rats, which may be related to the induction of inflammatory status (Jiang et al., 2015).

### 2.4 Prostanoids in cardiovascular systems

Prostaglandins (PGs) and thromboxanes (TXs) are collectively referred to as prostanoids. Prostanoids are a class of lipid mediators produced from AA through enzymatic metabolism. Under various physiological and pathological stimuli, AA is catalyzed by phospholipase A2 (PLA2), released from cell membrane phospholipids, and then converted into prostaglandin intermediates PGG2 and PGH under the activity of COX enzymes, which are then metabolized into various bioactive prostaglandins, including PGD2, PGE2, PGF2α, PGI2, TXA2, and others (Beccacece et al., 2023).

Among the different prostanoids, PGD2 is expressed in cells involved in immunity and inflammation (Beccacece et al., 2023). Both PGD2 and its main degradation product, 15-deoxy-∆-12, 14-Prostaglandin J2 (15d-PGJ2), play roles in inflammation resolution (Yousefnia et al., 2018). PGE2 is one of the most abundant prostaglandins and participates in all processes leading to inflammation (Nakanishi and Rosenberg, 2013). PGF2α is considered a vasoconstrictor of coronary arteries, associated with cardiac dysfunction and hypertrophy (Adams et al., 1998; Kuhn et al., 2015). PGI2 and TXA2 are considered the predominant prostaglandins of the cardiovascular system (Beccacece et al., 2023) produced by vascular endothelial cells and platelets, respectively, exerting opposite effects on vessels and platelets. PGI2 induces vasodilation and inhibits platelet aggregation (Ozen and Norel, 2017), TXA2 induces vasoconstriction and is a potent platelet agonist (Scridon, 2022). The balance between PGI2 and TXA2 is one of the key factors determining the homeostasis of the cardiovascular system. Clinical research shows that for every unit increase in the TXB2 (a metabolite of TXA2)/PGF1α (a downstream metabolite of PGI2) ratio, the likelihood of cardiovascular events increases (Caligiuri et al., 2017a).

Diseases such as thrombosis and atherosclerosis predominantly involve abnormal changes in prostanoids-type oxylipins, closely associated with the activation, adhesion, and aggregation of platelets. Atherosclerosis, a precursor to coronary artery disease and heart failure, involves lipid deposition and thrombus formation, leading to fibrous tissue proliferation and calcification (Tong et al., 2022). These processes thicken and harden the arterial walls, narrow the lumen, induce myocardial ischemia, and ultimately lead to heart failure. Research on oxylipins in atherosclerosis is limited, but increases in TXB2 levels and decreases in 6-keto-PGF1α have been noted (Zhang et al., 2013). Clinical studies have shown that oxylipins, including 11-dehydro-2, 3-dinor-TXB2 is closely associated with blood pressure status (Palmu et al., 2020). In hypertensive patients, elevated levels of TXs (TXA2) and reduced levels of prostanoids have also been observed (Jiang et al., 2015; Xie et al., 2019; Feugray et al., 2022). PGD2/PGE2 and 15d-PGJ2 are inversely correlated with CAD events (Chiang et al., 2022). Compared to healthy volunteers, CAD patients exhibit elevated levels of PGE2 (Theken et al., 2012). Similarly, prostanoids change post-myocardial infarction. Prostanoids like TXB2, TXA2, PGD2, PGE2, PGF2, and 15-Deoxy-PGJ2 rise, while 6-keto-PGF1α and PGI2 decrease (Cao et al., 2016; Li et al., 2016; Jiang et al., 2021; Zhi et al., 2021).

As the terminal stage of various cardiac diseases, heart failure is characterized by structural changes and functional impairments (Snipelisky et al., 2019). Abnormal oxylipins associated with heart failure reflect inflammatory responses, oxidative stress, myocardial fibrosis, and changes in cellular energy metabolism (González et al., 2018; Yin et al., 2019), with notable lipids such as PGE2 mediating inflammation, myocardial fibrosis, and cardiac cell apoptosis. Specifically, in terms of inflammation, prostaglandins like 15R-PGF2α, 11β-DHK-PGF2α, and PGE1 show high relevance to heart failure (Lau et al., 2023). As for myocardial fibrosis, the AA-COX pathway is essential for synthesizing prostanoids (e.g., PGI2, PGD2, PGE2, PGF2, and TXA2), representing the most extensively studied pathway in this area. Studies indicate that in heart failure models in rats, the expression of COX1 and COX2 are significantly upregulated, levels of 13, 14-dihydro-PGF2α, TXB2, PGE2, TXB2/6-keto-PGF1α rise significantly, while 6-keto-PGF1α decreases, concurrently with RAAS system activation, exacerbating the myocardial fibrosis process and inducing heart failure (Wang et al., 2015b).

### 2.5 Epoxyoctadecaenoic acids (EpOMEs) in cardiovascular systems

EpOMEs are oxides generated by LA via the action of CYP cyclooxygenase. Among the family of EpOMEs, 9, 10-EpOMEs have cardiac inhibitory effects (Sugiyama et al., 1987), and 12, 13-EpOMEs have a higher correlation with heart failure (Lau et al., 2023). Both 9, 10-EpOME and 12, 13-EpOME exhibit elevated levels in CAD patients (Theken et al., 2012). sEH converts EpOMEs to DiHOMEs. Both EpOMEs and DiHOMEs are cytotoxic, cardiotoxic, and can mediate inflammation and vasoconstriction (Hildreth et al., 2020). In CYP2C8-Tie2 mice, hearts perfused in the Langendorff mode with 9, 10-DiHOME or 12, 13-DiHOME showed a reduced recovery rate from reperfusion injury and increased coronary artery resistance (Edin et al., 2011; Bannehr et al., 2019). However, there are also studies supporting the beneficial effects of 12, 13-EpOMEs and 12, 13-DiHOME (Bannehr et al., 2019). In one study, cardiac perfusion of rats with LA, 12, 13-EpOMEs, and 12, 13-DiHOME resulted in a moderate increase in cardiac contraction observed within 10–20 min, with the perfusion effect of 12, 13-DiHOME lasting into the washout period. Accordingly, it is suggested that the impact of EpOMEs and DiHOMEs on cardiovascular function is still uncertain, and the specific effects may be related to other factors such as the dose of oxylipins and the environment.

### 2.6 Hydroxyoctadecadienoic acids (HODEs) in cardiovascular systems

HODEs are secondary oxidation products of LA, mainly formed through hydroxylation by CYP epoxygenases. The role of HODE in cardiovascular disease remains unclear, as the results of current studies are not consistent. Common HODEs include 9-HODE and 13-HODE. Both 9-HODE and 13-HODE can regulate oxidative stress and inflammation (Folcik and Cathcart, 1994; Krämer et al., 1996; Marmol et al., 1999; Belvisi and Mitchell, 2009). 9-HODE mediates alterations in pro-inflammatory markers associated with chronic inflammation. One study showed that 9-HODE had an inflammation-inducing effect and regulated Forkhead box O nuclear levels through the c-Jun N-terminal protein kinases pathway, linking fatty acid homeostasis, inflammation, and insulin resistance (Kwon et al., 2020). In contrast to 9-HODE, 13-HODE has anti-inflammatory effects (Marmol et al., 1999; Belvisi and Mitchell, 2009), with 13-HODE levels negatively correlated with vascular wall adhesiveness, inhibiting thrombus formation on damaged vascular walls and exerting numerous beneficial effects on cardiovascular health (Buchanan et al., 1991).

Dehydrogenation of HODE by dehydrogenase results in the formation of oxo-ODE. In nested case-control studies of CAD patients, 13-oxo-ODE appears to have a harmful effect (Chiang et al., 2022). The metabolic disorders of HODE and oxo-ODE often occur in acute cardiovascular diseases, such as myocardial infarction (MI) and reperfusion injury after myocardial infarction. However, it is worth noting that HODE and oxo-ODE do not have the same metabolic disturbances in the same disease, probably due to the variability of animal experiments and clinical trials. In clinical trials of myocardial infarction and post-myocardial infarction reperfusion injury, the levels of 9-HODE, 13-HODE, 9-oxo-ODE, and 13-oxo-ODE showed varying degrees of decline (Li et al., 2016; Solati et al., 2023). However, in rats with myocardial infarction, the levels of all these oxylipins were increased (Zhi et al., 2021).

## 3 Modulation of oxylipins in cardiovascular diseases by traditional Chinese herbal medicines

**FIGURE 2:**
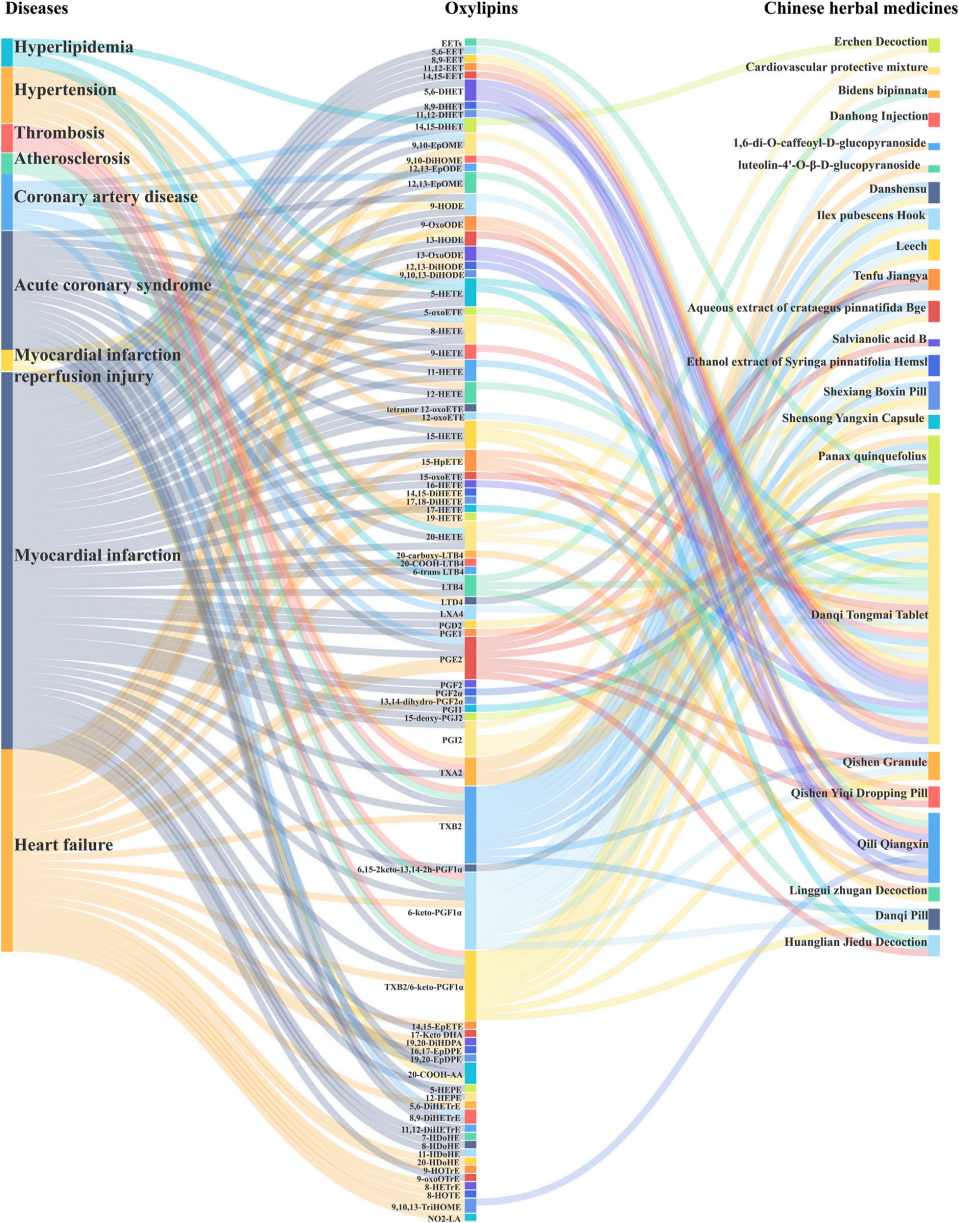
Disrupted Oxylipins in Cardiovascular Disease and Oxylipins Targets in Chinese Herbal Medicine. This Sangi diagram is used to demonstrate the oxylipin targets of cardiovascular disease and Chinese herbal medicine, providing a comprehensive overview of the regulation of oxylipins by Chinese herbal medicine in cardiovascular disease.

In China, several traditional Chinese medicine (TCM) formulations are commonly used as adjunct therapies for cardiovascular diseases, including the Xinyue Capsules and Danqi Tongmai Tablets, which are popular used in clinics (Zhi et al., 2021; Wang et al., 2023). Modern pharmacology has shown that these TCM formulations are beneficial for the treatment and prognosis of cardiovascular diseases. As research continues to deepen, the mechanisms by which TCM regulates oxylipins are being gradually revealed, especially in animal experiments and *in vitro* cell studies. Currently, the regulation of oxylipins in cardiovascular diseases by TCM mainly involves lipids such as EETs, HETEs, PGs, HODEs, and EpOMEs produced through the LOX, COX, and CYP pathways (refer to Table 3; Figure 2 for details).

**TABLE 3:** Summary of Chinese herbal medicines that Regulate Oxylipins mechanisms.

Diseases	Chinese herbal medicine	Main component(s)/constituent(s)	Oxylipins targets (↑/↓)	Reference
Hypercholesterolemia	Erchen Decoction	*Pinellia ternata* (Thunb.) Breit. [Araceae], *Poria cocos* (Schw.) Wolf. [Polyporaceae], *Citrus reticulata* Blanco. [Rutaceae], etc.	CYP: 14, 15-DHET↑, 20-HETE↓	Chen et al. (2022)
Hypercholesterolemia	Cardiovascular protective mixture	*Salvia miltiorrhiza* Bunge. [Lamiaceae], *Ligusticum chuanxiong* Hort. [Apiaceae], *Angelica sinensis* (Oliv.) Diels. [Apiaceae], etc.	COX: PGI2↑	Tu et al. (2003)
Hypercholesterolemia	Bidens bipinnata L. [Asteraceae]	—	LOX: LTB4↓	Wang et al. (2020b)
Hyperlipidemia	Danhong Injection	*Salvia miltiorrhiza* Bunge. [Lamiaceae], *Carthamus tinctorius* L. [Asteraceae]	COX: 6-keto-PGF1α↑, TXA2↓, PGE2↓ PGI2↑	Wang et al. (2013), Fan et al. (2018)
hypertension	Tengfu Jiangya	*Uncaria rhynchophylla* (Miq.) Miq. ex Havil or *Uncaria macrophylla* Wall or *Uncaria hirsuta* Havil or *Uncaria sinensis* (Oliv.) Havil or *Uncaria sessilifructus* Roxb. [Rubiaceae], *Raphanus sativus* L. [Brassicaceae]	COX: TXB2↓, PGE2↓ LOX: LTD4↓	Jiang et al. (2015)
Thrombosis	*Callicarpa nudiflora* Hook. and Arn. [Lamiaceae]	1,6-di-O-caffeoyl-D-glucopyranoside	COX: TXA2↓	Fu et al. (2017)
Thrombosis	*Callicarpa nudiflora* Hook. and Arn. [Lamiaceae]	luteolin-4′-O-β-D-glucopyranoside	COX: TXA2↓	Xu et al. (2018)
Thrombosis	*Salvia miltiorrhiza* Bunge. [Lamiaceae]	Danshensu	COX: TXB2↓, 6-keto-PGF1α↑ Ratio: TXB2/6-keto-PGF1α↓	Yu et al. (2014)
Thrombosis	*Ilex pubescens* Hook. and Arn. [Aquifoliaceae]	—	COX: TXB2↓, 6-keto-PGF1α↑ Ratio: TXB2/6-keto-PGF1α↓	Cao et al. (2018)
Atherosclerosis	*Whitmania pigra* Whitman or *Hirudo nipponica* Whitman or *Whitmania acranulata* Whitman. [Haemopidae]	—	COX: TXB2↓, 6-keto-PGF1α↑ Ratio: TXB2/6-keto-PGF1α↓	Jiang et al. (2019)
Atherosclerosis	*Crataegus Pinnatifida* Bge. var. *major* N. E. Br. [Rosaceae]	Aqueous extract of *Crataegus Pinnatifida* Bge. var. *major* N. E. Br. [Rosaceae]	COX: TXB2↓, 6-keto-PGF1α↑ Ratio: TXB2/6-keto-PGF1α↓	Zhang et al. (2013)
Coronary heart disease	*Salvia miltiorrhiza* Bunge. [Lamiaceae]	Salvianolic acid B	COX: PGE2↓	Wang et al. (2013)
Myocardial ischemia	*Syringa pinnatifolia* Hemsl. [Oleaceae]	Ethanol extract of *Syringa pinnatifolia* Hemsl	COX: TXB2↓, 6-keto-PGF1α↑ Ratio: TXB2/6-keto-PGF1α↓	Zhang et al. (2022)
Myocardial infarction	Shexiang Boxin Pill	*Moschus berezovskii* Flerov or *Moschus sifanicus* Przewalski or *Moschus moschiferus* Linnaeus. [Cervidae], *Panax ginseng* C. A. Mey. [Araliaceae], and *Liquidambar orientalis* Mill. [Hamamelidaceae R. Br.]	COX: TXB2↓, 6-keto-PGF1α↑ CYP: 20-HETE↑ Ratio: TXB2/6-keto-PGF1α↓	Huang et al. (2017)
Myocardial infarction	Shensong Yangxin Capsule	*Panax ginseng* C. A. Mey. [Araliaceae], *Nardostachys jatamansi* DC. [Caprifoliaceae], *Salvia miltiorrhiza* Bunge. [Lamiaceae] , etc.	COX: TXA2↓, PGI2↑	Jiang et al. (2021)
Myocardial infarction	*Panax quinquefolium* L. [Araliaceae]	Panax quinquefolius saponins	COX: TXA2↓TXB2↓, PGI2↑, 6-keto-PGF1α↑, 6, 15-2keto-13, 14-2h-PGF1α↑ CYP: EET↑, DHET↑ Ratio: TXB2/6-keto-PGF1α↓	Wang et al. (2023)
Acute myocardial infarction	Danqi Tongmai Tablet	Salvianolic acid extract and *Panax notoginseng* (Burk.) F. H. Chen. [Araliaceae]	Cox: PGD2↓, PGE2↓, TXB2↓ 15-deoxy-PGJ2↓, PGF2α↓, PGE1↑, PGI1↑, PGI2↑, 6-keto-PGF1α↑ Ratio: TXB2/6-keto-PGF1α↓ LOX: 5-HETE↓, 5-oxoETE↓, LTB4↓, 12-HETE↓, 12-oxoETE↓, 15-HETE↓, 15-oxoETE↓, 15-HpETE↓ LXA4↑ CYP: 8-HETE↓, 9-HETE↓, 11-HETE↓, 20- HETE↓ 5, 6-EET↑, 8, 9-EET↑, 11,12-EET↑, 14, 15-EET↑, 5, 6-DHET↑, 8, 9-DHET↑, 11, 12-DHET↑, 16-HETE↑, 17-HETE↑ Oxidation: 9-HODE↓, 13-HODE↓, 9-oxoODE↓, 13-oxoODE↓	Zhi et al. (2021)
Acute myocardial infarction	Qishen Granule	*Astragalus membranaceus* (Fisch.) Bge.var.*mongholicus* (Bge.) Hsiao or *Astragalus membranaceus* (Fisch.) Bge. [Fabaceae], *Salvia miltiorrhiza* Bunge. [Lamiaceae], *Scrophularia ningpoensis* Hemsl. [Scrophulariaceae], etc.	COX: TXB2↓, PGE2↓, 6-keto-PGF1α↑ Ratio: TXB2/6-keto-PGF1α↓	Li et al. (2016)
Heart failure, Acute myocardial infarction	Qishen Yiqi Dropping Pill	*Astragalus membranaceus* (Fisch.) Bge.var.*mongholicus* (Bge.) Hsiao or *Astragalus membranaceus* (Fisch.) Bge. [Fabaceae], *Salvia miltiorrhiza* Bunge. [Lamiaceae], *Panax notoginseng* (Burk.) F. H. Chen. [Araliaceae], etc.	LOX:15-HpETE↓, 15-HETE↓ COX: PGE2↓	Wang et al. (2015a)
Heart failure	Qili Qiangxin	*Panax ginseng* C. A. Mey. [Araliaceae], *Astragalus membranaceus* (Fisch.) Bge.var.*mongholicus* (Bge.) Hsiao or *Astragalus membranaceus* (Fisch.) Bge. [Fabaceae], *Aconitum carmichaelii* Debeaux. [Ranunculaceae] , etc.	CYP: 9, 10-EpOME↓, 12, 13-EpOME↓, 9, 10-DiHOME↓, 5, 6-DHET↑ LOX: 9-OxoODE↓, 13-OxoODE↓, 9, 10, 13-TriHOME↓, 12, 13-DiHODE↓, 20-carboxy-LTB4↓, 5,6-DHET↑	Fu et al. (2018)
Heart failure	Linggui zhugan Decoction	*Poria cocos* (Schw.) Wolf. [Polyporaceae], *Cinnamomum cassia* Presl. [Lauraceae], *Atractylodes macrocephala* Koidz. [Asteraceae], etc.	LOX: 15-HpETE↓, 15-HETE↓	Wang et al. (2020a)
Heart failure	Danqi Pill	*Salvia miltiorrhiza* Bunge. [Lamiaceae], *Panax notoginseng* (Burk.) F. H. Chen. [Araliaceae]	COX: TXB2↓, 6-keto-PGF1α↑ Ratio: TXB2/6-keto-PGF1α↓	Wang et al. (2014)
—	Huanglian Jiedu Decoction	*Coptis chinensis* Franch. [Ranunculaceae], *Scutellaria baicalensis* Georgi. [Lamiaceae], *Phellodendron chinense* Schneid. [Rutaceae], etc.	LOX: LTB4↓, 5-HETE↓ COX: PGE2↓	Yuan et al. (2020)

### 3.1 Regulation of EET by traditional Chinese herbal medicines

TCM formulations, including Xinyue Capsules, Danqi Tongmai Tablets, and Erchen Decoction, regulate four abnormal types of EETs involved in cardiovascular diseases. Xinyue Capsules, a TCM used for treating cardiovascular diseases, primarily consist of Panax quinquefolius saponins (PQS) and are widely used as a supplementary antiplatelet therapy. Recent pharmacological studies have shown that PQS, in combination with antiplatelet drugs, have a synergistic protective effect on platelet adhesion to endothelial cells and reduce the bleeding risk associated with antiplatelet drugs (Wang et al., 2023). This effect is likely due to the activation of the AA-CYP-EET pathway, leading to increased EET synthesis, reduced platelet adhesion and aggregation, and elevated levels of 8, 9-DHET and 11, 12-DHET, thereby ameliorating the cardiac dilation observed in MI rats (Wang et al., 2023).

Danqi Tongmai Tablet, a TCM formula for treating blood stasis-type stable angina pectoris, mainly composed of Salvianolic acid extract and *Panax notoginseng* (Burk.) F. H. Chen. [Araliaceae], can increase the levels of 5, 6-EET, 8, 9-EET, 14, 15-EET, 5, 6-DHET, and 11, 12-DHET in the plasma of AMI rats through the CYP2C/2J pathway and downregulate pro-inflammatory cytokines, significantly improving the inflammatory state of AMI rats. The modulation of 5, 6-EET and 8, 9-EET by Danqi Tongmai Tablet at a concentration of 130 mg/kg was the most pronounced, revealing a dose-dependent regulation of arachidonic acid metabolites EET by Danqi Tongmai Tablet (Zhi et al., 2021).

The Erchen Decoction, composed of *Pinellia ternata* (Thunb.) Breit. [Araceae], *Poria cocos* (Schw.)Wolf. [Polyporaceae], *Citrus reticulata* Blanco. [Rutaceae], etc. (names based on the Chinese Pharmacopoeia and http://mpns.kew.org/mpns-portal/), has the effects of drying dampness and resolving phlegm, regulating qi, and harmonizing the middle. It is often used in the treatment of clinical lipid metabolism disorders (Li S. et al., 2020; Zhang L. et al., 2023). In the formula-disease correspondence group, the concentration of 14, 15-DHET was significantly increased, indicating that Erchen Decoction protected the vascular endothelium of mice with dyslipidemia and stasis syndrome by increasing serum 14, 15- DHET (Chen et al., 2022). Similarly, Qili Qiangxin Capsule, a TCM formula mainly composed of *Panax ginseng* C. A. Mey. [Araliaceae], *Astragalus membranaceus* (Fisch.) Bge. var.*mongholicus* (Bge.) Hsiao or *Astragalus membranaceus* (Fisch.) Bge. [Fabaceae] (Usually using *Astragalus membranaceus* (Fisch.) Bge. var.*mongholicus* (Bge.)Hsiao), *Aconitum carmichaelii* Debeaux. [Ranunculaceae], etc., could increase levels of 5, 6-DHET through the AA-CYP450 pathway, regulating inflammatory responses induced by heart failure (Fu et al., 2018).

### 3.2 Regulation of HETEs by traditional Chinese herbal medicines

Current research indicates that TCM regulation of HETEs primarily focuses on 5-, 12-, 15-, 16-, 17-, and 20-HETE. As previously mentioned, 5-, 12-, and 15-HETE all have harmful effects on the cardiovascular system. The main functions of 16-HETE include promoting vasodilation and inhibiting inflammation mediated by neutrophil adhesion. In AMI model rats, Danqi Tongmai Tablet can elevate levels of 16-HETE and 17-HETE through the CYP pathway and reduce levels of 5-, 12-, 15-HETE, and 5-, 15-oxoETE through the LOX pathway.

The exact role of 20-HETE in the cardiovascular system needs further exploration. Current research evidence indicates the beneficial effects of 20-HETE but also emphasizes its harm to the cardiovascular system. In dyslipidemic and stasis syndrome model mice (n-5C57BL/6J mice and n-30 apolipoprotein E knockout mice), the serum concentration of 20-HETE was reduced by Erchen Decoction, and the effect of Erchen Decoction on 20-HETE was also related to the prescription-syndrome correspondence. When the prescription-syndrome did not correspond, Erchen Decoction had no significant regulatory effect on 20-HETE compared to the model group (Chen et al., 2022). However, some studies support the beneficial effects of 20-HETE, highlighting its ability to promote endothelial cell proliferation via the VEGF pathway (Chen et al., 2012). Some TCMs, like Shexiang Baoxin Pill, a pill made from *Moschus berezovskii* Flerov or *Moschus sifanicus* Przewalski or *Moschus moschiferus* Linnaeus. [Cervidae] (Usually using *M. berezovskii* Flerov), *Panax ginseng* C. A. Mey. [Araliaceae], and *Liquidambar orientalis* Mill. [Hamamelidaceae R. Br.], etc., commonly used in China for the clinical treatment of angina. Shexiang Baoxin Pill can increase levels of 20-HETE and endothelial progenitor cells (EPCs) in rats with myocardial infarction, along with increasing VEGF expression. Conversely, HET0016 (a 20-HETE synthesis inhibitor) could partially weaken the effects of Shexiang Baoxin Pill on EPCs, VEGF, and angiogenesis. These results strongly suggest that the effects of Shexiang Baoxin Pill on angiogenesis in myocardial infarction are mediated by promoting 20-HETE-induced mobilization of EPCs and VEGF expression (Huang et al., 2017).

### 3.3 Regulation of LTs by traditional Chinese herbal medicines

Danqi Tongmai Tablet can reduce LTB4 levels through the LOX5 pathway, significantly downregulate pro-inflammatory cytokines, and alleviate the inflammatory status of AMI model rats (Zhi et al., 2021). In spontaneously hypertensive rats, LTD4 levels were elevated, and the Chinese herbal formula Tengfu Jiangya Tablet, main constituents are *Uncaria rhynchophylla* (Miq.) Miq. ex Havil or *Uncaria macrophylla* Wall or *Uncaria hirsuta* Havil or *Uncaria sinensis* (Oliv.) Havil or *Uncaria sessilifructus* Roxb. [Rubiaceae] (Usually using *Uncaria rhynchophylla* (Miq.) Miq. ex Havil) and *Raphanus sativus* L. [Brassicaceae], with the effects of lowering blood pressure) could lower leukotriene LTD4 levels through AA metabolism, suggesting that the interaction between inflammation and hypertension could be one of the potential mechanisms by which leukotrienes exert cardiovascular protective effects (Jiang et al., 2015).

### 3.4 Regulation of prostaglandins by traditional Chinese herbal medicines

Various herbal formulas have regulatory effects on PGs/TXs oxylipins by COX pathway. For example, Danqi Tongmai Tablet can restore AA metabolism disorder, downregulate metabolic levels of PGD2, PGE2, PGF2α, 15-d-PGJ2, TXB2, and upregulate levels of 6-keto-PGF1α, PGE1, and PGI1 in AMI rats (Zhi et al., 2021). Danhong Injection, a Chinese herbal injection made from *Salvia miltiorrhiza* Bunge. [Lamiaceae] and *Carthamus tinctorius* L. [Asteraceae], is commonly used in the treatment of occlusive cardiovascular diseases (Zhang et al., 2019). It has been shown that Danhong Injection could relax aortic vessels, increase COX mRNA expression, raise 6-keto-PGF1α levels, and promote PGI2 release *in vitro*. Moreover, in hyperlipidemic model rats, Danhong Injection could increase PGE mRNA and 6-keto-PGF1α expression and decrease TXA2 levels, thereby inhibiting platelet aggregation (Wang et al., 2013; Fan et al., 2018). Salvianolic acid, the main component of traditional Chinese herbal medicine *Salvia miltiorrhiza* Bunge. [Lamiaceae], can reduce PGE2 levels and inhibit lipid peroxidation through the AA pathway (Li Y. P. et al., 2020).

In TCM formulations, Qishen Yiqi Dripping Pill, a TCM formula for treating blood stasis-type stable angina pectoris, mainly composed of *Astragalus membranaceus* (Fisch.) Bge. var.*mongholicus* (Bge.) Hsiao or *Astragalus membranaceus* (Fisch.) Bge. [Fabaceae] (Usually using *Astragalus membranaceus* (Fisch.) Bge. var.*mongholicus* (Bge.) Hsiao, *Salvia miltiorrhiza* Bunge. [Lamiaceae], *Panax notoginseng* (Burk.) F. H. Chen. [Araliaceae], etc., can lower NF-κB, COX2, and PGE2 receptor levels, alleviating the inflammatory state (Wang et al., 2015b). Under pathological conditions, inflammation stimulates the production of PGE2 through the COX2/mPGES-1 (membrane-associated prostaglandin E2 synthase 1) pathway, leading to macrophage activation in plaques and matrix metalloproteinase (MMP)2 and MMP9 via the cAMP-dependent pathway. MMPs can degrade the extracellular matrix in the fibrous cap, reducing its components, thinning the fibrous cap, making plaques unstable and prone to rupture, leading to acute ischemic events (Mezzetti, 2005; Sun and Li, 2018). In addition, PGE2 can activate G protein-coupled receptors EP2 and EP4, both of which are associated with vasodilation, stimulation of inflammation, and cardiac hypertrophy (Torres et al., 2015; Sun and Li, 2018; Wang et al., 2019). EP4 is also one of the most important receptors in human inflammation-related diseases (Takayama et al., 2002; Tang et al., 2012), playing a significant role in PGE2-dependent MMP expression. In ankylosing spondylitis patients with concomitant cardiovascular diseases, EP4 expression is much higher than in ankylosing spondylitis patients without clinical manifestations (Cipollone et al., 2005). Thus, it can be inferred that COX2, PGE2, and its receptors EP2, EP4, MMP play significant roles in cardiovascular events. Qishen Yiqi Dripping Pill downregulates AA levels through the AA-COX1/COX2-PGE2 pathway, inhibiting PGE2-mediated apoptosis, regulating PGE2 downstream metabolites EP2 and EP4, reducing MMP2 and MMP9 production, and downregulating p53 and FasL protein. Compared to the model group, the myocardial apoptosis rate in the Qishen Yiqi Dripping Pill group rats was decreased (Wang et al., 2015b; 2015a).

The cardiovascular protective mixture, a TCM formula made from *Salvia miltiorrhiza* Bunge. [Lamiaceae], *Ligusticum chuanxiong* Hort. [Apiaceae], and nine other blood-activating and stasis-removing herbs, can activate the proliferation of vascular endothelial cells *in vitro*, thereby promoting the synthesis and secretion of PGI2 and playing a protective role in the vascular endothelium (Tu et al., 2003). In the MI model established by left coronary artery ligation, the expression level of PGI2 mRNA decreased and TXA2 increased compared to the MI group. Shensong Yangxin Capsule (main constituents: *Panax ginseng* C. A. Mey. [Araliaceae], *Nardostachys jatamansi* DC. [Caprifoliaceae], *Salvia miltiorrhiza* Bunge. [Lamiaceae], etc., with the effects of benefiting qi and nourishing yin, activating blood and dredging collaterals). could increase plasma PGI2 mRNA levels in MI rabbits, reduce TXA2 levels, thereby balancing vasoconstriction and dilation (Jiang et al., 2021). Among the single herbs in Chinese medicine, *Callicarpa nudiflora* Hook. and Arn. [Lamiaceae], a herb that dispels blood stasis, reduces swelling, and stops bleeding, has an extract, 1,6-di-O-caffeoyl-β-D-glucopyranoside, that has been found to resist P2Y12 and TP2 receptors (prostaglandin-like), thereby inhibiting TXA2 synthesis (Fu et al., 2017). In MI model rats, Panax quinquefolius saponins combined with dual antiplatelet therapy (PQS + DAPT) significantly increased PGI2 synthesis, reduced platelet activation and thrombogenesis agonist TXA2 synthesis and TXB2 levels, while plasma levels of 6-keto-PGF1α increased tenfold, and 6,15-2keto-13,14-2H-PGF1α levels increased threefold. This suggests that the improvement in platelet inhibition by PQS + DAPT may be partly due to the upregulation of AA/PGI2 and the downregulation of AA/TXA2 metabolism (Wang et al., 2023). The downregulation of TXA2 metabolism by PQS + DAPT could be the basis for the enhanced anti-aggregation effect of the combined therapy, possibly helping to reduce cardiovascular events to some extent. Additionally, PGI2 can counteract the thrombotic properties of TXA2, further inhibiting platelet activation. Further analysis found that PGI2 is co-regulated by COX1 and COX2 produced by endothelial cells, which possess vasodilatory properties. In endothelial cells, the use of DAPT alone or in combination with PQS does not affect the expression of COX1 protein; however, it elicits distinct effects on the expression and biological activity of COX2 protein. When DAPT was used alone, COX2 protein expression decreased, and activity declined, whereas PQS + DAPT had no inhibitory effect on COX2 protein expression, and at the same time, COX2 protein activity was enhanced. TXA2 is synthesized in platelets through the COX1 pathway, possessing vasoconstrictive properties. In platelets, compared to the use of DAPT alone, PQS + DAPT showed no significant differences in the regulation of COX1 and COX2 expression and activity. Therefore, compared to the inhibitory effect of the platelet COX1/TXA2 pathway, the regulatory effect of the combined application of PQS with DAPT on the endothelial cell COX2/PGI2 pathway is more optimal (Wang et al., 2023).

*Syringa pinnatifolia* Hemsl. [Oleaceae] is a Chinese herb mainly produced in Mongolia, China, which has the effect of moving qi and relieving pain and is often made into powder or Chinese medicine formulations for the treatment of myocardial ischemia (Zhang et al., 2022). The peeled extract of *Syringa pinnatifolia* Hemsl. [Oleaceae] downregulated the expression of COX1 and COX2 expression in myocardial ischemia model mice (C57BL/6 mice). Specifically, the strongest inhibitory effects on COX2 and COX1 were observed when the drug concentrations of the extract of *Syringa pinnatifolia* Hemsl. [Oleaceae] were at 20–80 mg/kg and 80 mg/kg, respectively. In addition, *Syringa pinnatifolia* Hemsl. [Oleaceae] pretreatment slightly increased plasma levels and reduced TXB2 production, while the opposite was observed in protein homogenates. Interestingly, the 6-keto-PGF1α/TXB2 ratio in the *Syringa pinnatifolia* Hemsl. [Oleaceae] pretreatment group was strongly dose-dependent, approaching the observed values in the myocardial ischemia sham surgery group, and was significantly superior to the positive drug pretreatment group (Cao et al., 2016).

The balance between 6-keto-PGF1α and TXB2 maintains the homeostasis of the cardiovascular system. Some blood-activating Chinese medicines like aqueous extract of *Crataegus Pinnatifida* Bge. var. *major* N. E. Br. [Rosaceae], Danshensu, Leech powder, the formulas Qishen Granule (main constituents: *Astragalus membranaceus* (Fisch.) Bge. var.*mongholicus* (Bge.) Hsiao or *Astragalus membranaceus* (Fisch.) Bge. [Fabaceae] (Usually using *Astragalus membranaceus* (Fisch.) Bge. var.*mongholicus* (Bge.) Hsiao, *Salvia miltiorrhiza* Bunge. [Lamiaceae], *Scrophularia ningpoensis* Hemsl. [Scrophulariaceae], etc.), Shexiang Boxin pill, and Danqi Tablet (main constituents: *Salvia miltiorrhiza* Bunge. [Lamiaceae] and *Panax notoginseng* (Burk.) F. H. Chen. [Araliaceae]) have mechanisms similar to the peeled extract of *Syringa pinnatifolia* Hemsl. [Oleaceae]. These extracts are all capable of lowering plasma TXB2 levels, increasing plasma 6-keto-PGF1α levels, maintaining a relatively stable 6-keto-PGF1α/TXB2 ratio, reducing lipid peroxidation levels (Zhang et al., 2013; Wang et al., 2014; Yu et al., 2014; Li et al., 2016; Li J. et al., 2020; Huang et al., 2017; Jiang et al., 2019).

### 3.5 Regulation of EpOMEs by traditional Chinese herbal medicines

In rats with heart failure, levels of LA-produced 9, 10-EpOME and 12, 13-EpOME were elevated, and Qili Qiangxin Capsule reduced these oxylipin levels while maintaining stable levels of 9, 10-DiHOME, thereby controlling cardiac hypertrophy and inflammation associated with heart failure (Fu et al., 2018).

### 3.6 Regulation of HODEs by traditional Chinese herbal medicines

TCM has been shown to regulate two common types of HODEs. Interestingly, in a study involving Danqi Tongmai Tablets, these tablets significantly reduced the levels of the pro-inflammatory lipid 9-HODE and the anti-inflammatory lipid 13-HODE in rats with myocardial infarction compared to those in acute myocardial infarction and sham operation groups. The levels of their downstream metabolites, 9-OxoODE and 13-OxoODE, were also significantly lowered (Zhi et al., 2021). However, the inflammation status of MI in the Danqi Tongmai Tablet group was still improved, suggesting that Danqi Tongmai Tablet may have a stronger regulatory effect on anti-inflammatory oxylipins, though further research is needed to confirm these results. Similarly, in rats with heart failure, Qili Qiangxin Capsule significantly lowered downstream metabolites of 9-HODE and 13-HODE, such as 9-OxoODE, 13-OxoODE, 12-, 13-DiHODE, and 9-, 10-, 13-TriHOME (Fu et al., 2018), suggesting a regulatory mechanism for HODE metabolites similar to that of Danqi Tongmai Tablet.

### 3.7 Effects of Chinese herbal medicine on oxidized lipogenesis substrates and related enzymes

Currently, in the research of TCM in treating cardiovascular diseases, some studies have preliminarily revealed the effects of TCM on upstream substrates for oxylipin generation and related enzymes (including PLA2 and COX), such as the formula Xinkeshu Capsules (main constituents: *Salvia miltiorrhiza* Bunge. [Lamiaceae] and *Pueraria lobata* (Willd.) Ohwi. [Fabaceae], used for common cardiovascular diseases such as coronary heart disease, angina, hypertension, arrhythmia, and hyperlipidemia), Huanglian Jiedu Tang (main constituents: *Coptis chinensis* Franch. [Ranunculaceae], *Scutellaria baicalensis* Georgi. [Lamiaceae], *Phellodendron chinense* Schneid. [Rutaceae], etc., commonly used for febrile diseases), and Yixin Shu (main constituents: *Salvia miltiorrhiza* Bunge. [Lamiaceae], *Panax ginseng* C. A. Mey. [Araliaceae], *Ophiopogon japonicus* (L.f). Ker Gawl. [Liliaceae], etc., commonly used to treat coronary heart disease).

The mechanism of Xinke Shu Capsules in treating myocardial infarction involves inhibiting fatty acid β-oxidation, specifically manifested as reversing phospholipase A2ⅡA and regulating the levels of LA and AA in myocardial tissue (Sun et al., 2021). Huanglian Jiedu Decoction and Yixin Shu can restore AA levels in rats with heart failure (Xu et al., 2019; Yuan et al., 2020).

The COX/LOX pathway is an important route for LA and AA to be converted into oxylipins. Studies have shown that some Chinese medicinal herbs can regulate COX and LOX in animals with cardiovascular disease models, thereby improving myocardial injury and protecting the heart. Danshen Dripping Pill downregulates AA metabolism through the AT1-mediated PLA2-COX2/5-LOX metabolic pathway, inhibits RAAS system activation and MMPs expression, and thus inhibits myocardial fibrosis in myocardial failure rats (Zhang et al., 2018). In coronary heart disease models, Danqi pill downregulates the expression of PLA2, COX2, NF-κB on the inflammatory pathway, significantly upregulating PPARα levels on both gene and protein expression levels (Chang et al., 2016). Danqi Tongmai Tablet and Sanhuang Xiexin Decoction (A decoction composed of *Rheum palmatum* L or *Rheum tanguticum* Maxim. ex Balf or *Rheum officinale* Baill. [Polygonaceae] (Usually using *Rheum officinale* Baill), *Coptis chinensis* Franch. [Ranunculaceae], *Scutellaria baicalensis* Georgi. [Lamiaceae], and has the effects of purging fire, detoxifying, and relieving heat). Affected the gene and protein expression levels of COX mRNA, but with different effects. In the myocardial cell oxidative-glucose deprivation/reoxygenation model, Danqi Tongmai Tablet can increase COX2 mRNA, and reduce ALOX5 mRNA levels, alleviating oxidative damage in myocardial cells; in the atherosclerosis model, Sanhuang Xiexin Decoction downregulates COX2 gene expression and protein levels (Wang et al., 2011; Zhi et al., 2021).

The CYP pathway is another route for the conversion of polyunsaturated fatty acids into oxylipins, and many studies have demonstrated the regulatory effects of Chinese medicinal herbs on a variety of CYP enzymes, especially the CYP450 metabolic pathway (Gu et al., 2014; Wang et al., 2018). The cardiovascular protective effect of *Pueraria lobata* (Willd.) Ohwi. [Fabaceae] may be related to the inhibition of CYP2B6, CYP2C9, and CYP3A4 enzymes (Gu et al., 2014). EETs can inhibit tissue factor TF expression and prevent thrombosis, which is related to the activation of the PI3K/AKT pathway to inhibit NF-κB nuclear translocation and target CYP cyclooxygenase (Luo et al., 2023). *Salvia miltiorrhiza* Bunge. [Lamiaceae], a commonly used TCM for treating cardiovascular diseases with blood-activating and stasis-resolving effects, has Tanshinone IIA as its main component. Data mining revealed its potential therapeutic targets might lie in CYP450 3A4, CYP450 A1, and NF-κB1, which may exert an anti-inflammatory and cardiovascular protective effect (Chen, 2015). Ophiopogon D, the main pharmacologically active component of Shenmai Injection, has been used to prevent and treat cardiovascular diseases. Ophiopogon D can alleviate myocardial hypertrophy and inflammation through the CYP450 2J3-NF-κB pathway (Wang et al., 2018).

Most of the Chinese herbal medicines mentioned above are adjunctive therapeutic drugs for CVDs and have good therapeutic effects. Given the important role of oxylipins in CVDs, it is necessary to analyze the regulatory effect of traditional Chinese herbal medicine on oxylipins in CVDs using oxylipins as a therapeutic target. Our review found that traditional Chinese herbal medicine has regulatory effects on the oxylipins’ substrate, oxylipins, and downstream metabolites of oxylipins, indicating the enormous potential of traditional Chinese herbal medicine in the treatment of cardiovascular diseases. Further exploration is needed to explore the regulatory effects of more Chinese herbal medicines on oxylipins.

## 4 Discussion and perspectives

From the perspective of Chinese medicine involved in regulating oxylipins, we have conducted a frequency analysis of the involved Chinese medicines and found that the four most frequently mentioned are *Salvia miltiorrhiza* Bunge. [Lamiaceae] (Danshen in Chinese), *Panax notoginseng* (Burk.) F. H. Chen. [Araliaceae] (Sanqi in Chinese), *Panax ginseng* C. A. Mey. [Araliaceae] (Renshen in Chinese), and *Astragalus membranaceus* (Fisch.) Bge. var.*mongholicus* (Bge.) Hsiao or *Astragalus membranaceus* (Fisch.) Bge. [Fabaceae] (Huangqi in Chinese) (details are provided in the Supplementary Appendix. We have previously mentioned that AA can produce oxylipins such as prostanoids through the COX pathway. Prostanoids are closely related to platelet aggregation, endothelial cell adhesion, and vascular permeability. Some studies have shown that AA-COX-prostanoids are one of the most extensively studied pathways in the COX pathway, closely related to myocardial fibrosis. *Salvia miltiorrhiza* Bunge. [Lamiaceae] is a traditional Chinese herb that promotes blood circulation and improves cardiac fibrosis. In the systematic evaluation of the bioactive components of *Salvia miltiorrhiza* Bunge. [Lamiaceae] in the treatment of thrombotic diseases, Tanshinone, and Salvianolic acids are considered the main active components, including but not limited to Salvianolic Acid B, Tanshinone IIA, and Danshensu (Wei et al., 2023). From Table 2, we know that current research on Chinese medicine has revealed the regulatory effects of Danshensu and Salvianolic acids on oxylipins. Danshensu and Salvianolic acids target the AA-COX-prostanoids signaling pathway in thrombosis and coronary heart disease, which to some extent explains the anti-fibrotic effect of *Salvia miltiorrhiza* Bunge. [Lamiaceae]. Moreover, we also found that frequently used Chinese medicines *Panax notoginseng* (Burk.) F. H. Chen. [Araliaceae] and *Panax ginseng* C. A. Mey. [Araliaceae] belong to the same genus and have similar active components such as Ginsenoside Rg3 (Wang et al., 2022). Modern pharmacology shows that Ginsenoside Rg3 has antioxidant, anti-inflammatory, antihypertensive, and myocardial ischemia-reperfusion injury preventive effects, which may explain why different Chinese medicines have similar cardiovascular effects in regulating oxylipins (Liu et al., 2020; Wang et al., 2022). Furthermore, this leads us to further speculate that components such as Salvianolic acids and Ginsenoside Rg3, representative of Chinese medicine, may be effective tools for regulating oxylipins, awaiting further research confirmation.

Furthermore, from the current research on TCM, we find that the study of Chinese medicine regulating lipid oxidation not only involves lipid oxidation itself but also its upstream and downstream metabolites. Upstream metabolites of lipid oxidation, such as AA, LA, cPLA, LOX, etc., are closely related to the production of oxylipins and are themselves associated with platelet activation and other cardiovascular risk factors (Yeung et al., 2014), which may play a coordinated role in regulating cardiovascular function with oxylipins. However, it is worth mentioning that the upstream regulatory mechanism of oxylipins usually involves signal cell transduction, and genetic and epigenetic factors in addition to related enzymes and substrates. Various signaling pathways that activate phospholipases can increase the release of free fatty acids from cell membranes, thereby providing substrates for oxylipin synthesis. Hormones, growth factors, and cytokines are examples of signaling molecules that can modulate enzyme expression and activity in oxylipin pathways. Genetic polymorphisms and mutations in genes encoding oxylipin-synthesizing enzymes can significantly affect their expression and function. Additionally, epigenetic modifications such as DNA methylation and histone acetylation can alter gene expression in response to environmental signals. However, there is currently no research on signal cell transduction and genetic correlation in current studies. Looking at the downstream metabolites of oxylipins, they may involve other pathways such as myocardial cell apoptosis, inflammation, and myocardial fibrosis. For example, PGE2 is produced by AA through COX metabolism and has pro-inflammatory and apoptotic effects. The specific mechanism may be related to PGE2-mediated P53 and FasL. TCM has a regulatory effect on the AA-COX-prostanoids-P53/FasL pathway in cardiovascular diseases. For instance, Qishen Yiqi Drop Pills improve myocardial cells by reducing the expression of COX and PGE2 receptors, downregulating PGE2-mediated P53 and FasL proteins; similarly, there is also the PPAR inflammation pathway, where Chinese medicine Danqi Pill can regulate the PPARα/NF-kB signaling pathway related to inflammation, improving the inflammation state mediated by AA. In addition, a large number of oxylipins like 15-HETE, PGJ2, etc., seem to be able to activate PPAR (Chen et al., 2003; Li J. et al., 2019), thereby regulating platelet activation. From the perspective of upstream and downstream metabolites, the mechanism of action of oxylipins in TCM treatment of cardiovascular diseases needs further research to fill the research gap.

Currently, significant progress has been made in the study of the mechanisms by which TCM regulates oxylipins in cardiovascular diseases, yet certain shortcomings persist: (1) Oxylipins have diverse and complex chemical structures, making their detection and analysis methods particularly important. Traditional laboratory methods, such as UV visible and fluorescence spectrophotometry, nuclear magnetic resonance, chemiluminescence analysis, immunoassay, etc., have made some progress in the characterization and quantification of oxidized lipids, but they are very limited (Li L. et al., 2019; Kodali et al., 2020). In recent years, with the advancement of mass spectrometry technology, research on oxylipins in biological samples has been promoted. The combination of mass spectrometry and liquid chromatography can selectively identify individual compounds or compound groups with common characteristics, greatly improving the sensitivity and specificity of detection (Spickett and Pitt, 2015). Thus far, over 100 oxylipins have been identified. Regrettably, most of the experimental techniques employed in the study of TCM regulation of oxylipins still rely on the ELISA method, with only a few studies utilizing lipidomics techniques. Future research in TCM needs to adapt faster to technological developments, selecting new and appropriate lipid oxidation technologies for detection based on the focused lipid oxidation categories, which is conducive to obtaining consistent, reproducible, and reliable lipid oxidation omics results. Additionally, it should be noted that there are still some shortcomings in the current TCM studies using oxidative lipidomics techniques. These studies usually only focus on oxygenases related to inflammation and platelets, rather than the full spectrum. The lipid profiles used in TCM research include up to 71 oxylipins (Fu et al., 2018), while others include 38 oxylipins (Zhi et al., 2021), which is not conducive to a comprehensive revelation of TCM’s regulatory mechanisms and its cardiovascular effects. For instance, current research reveals a predominant focus on the AA-COX-prostanoids pathway in most TCM studies, a focus that may reflect researchers’ subjective bias. Thus, incorporating the full spectrum of oxylipins in TCM research is imperative. (2) Studies on the regulation of oxylipins by TCM primarily focus on metabolites produced by ω-6 PUFA (AA, LA), with less attention to those derived from ω-3 PUFA. The reason might be that the cardiovascular effects of most ω-3 PUFA-derived oxylipins remain unclear, although research has shown that the risk of cardiovascular diseases is reduced after intake of ω-3 PUFAs and their derivatives. Still, the specific cardiovascular effects of these oxylipins require further study to clarify their roles and regulatory mechanisms in cardiovascular diseases. (3) Current studies on the abnormal regulation of oxylipins in cardiovascular diseases by TCM are primarily based on animal experiments, which yield relatively uniform results, but there is a lack of related clinical studies, particularly large-scale clinical evidence. It remains uncertain whether the regulatory effects of Chinese herbal medicine on oxylipins in cardiovascular patients mirror those observed in animal studies. Also, further research is needed to investigate the impact of Chinese herbal monomers or individual herbs on oxylipins in cardiovascular disease. We also urge more researchers to focus on the significant role of oxylipins in cardiovascular disease and to conduct large-scale, long-term, high-quality clinical studies on Chinese herbal medicine. Such efforts would provide clearer insights into the regulatory effects and safety of Chinese herbal medicine on oxylipins, enhance the robustness of the data, and increase the potential for broader clinical application.
