# Supplementary material for: Oxylipins as therapeutic indicators of herbal medicines in cardiovascular diseases: a review
Source: Front Pharmacol. 2024 Dec 19;15:1454348. doi: 10.3389/fphar.2024.1454348 (PMC11693728; doi:10.3389/fphar.2024.1454348)
Supplement: Supplementary file 1 [file DataSheet1.docx]

Appendix: Frequency Chart of Traditional Chinese Herbal Medicine for Regulating Oxylipins (Top 14 Flavors)
